# Supplemental Insulin-Like Growth Factor-1 and Necrotizing Enterocolitis in Preterm Pigs

**DOI:** 10.3389/fped.2020.602047

**Published:** 2021-02-04

**Authors:** Kristine Holgersen, Xiaoyan Gao, Rangaraj Narayanan, Tripti Gaur, Galen Carey, Norman Barton, Xiaoyu Pan, Tik Muk, Thomas Thymann, Per Torp Sangild

**Affiliations:** ^1^Comparative Pediatrics and Nutrition, Department of Veterinary and Animal Sciences, Faculty of Health and Medical Sciences, University of Copenhagen, Frederiksberg, Denmark; ^2^Department of Neonatology, Southern Medical University Affiliated Maternal & Child Health Hospital of Foshan, Foshan, China; ^3^Takeda, Cambridge, MA, United States; ^4^Department of Neonatology, Rigshospitalet, Copenhagen, Denmark; ^5^Department of Pediatrics, Odense University Hospital, Odense, Denmark

**Keywords:** IGF-1, infant, intestine, preterm birth, gut, newborn, growth restriction, fetus

## Abstract

**Background:** Recombinant human IGF-1/binding protein-3 (rhIGF-1/BP-3) is currently tested as a therapy in preterm infants but possible effects on the gut, including necrotizing enterocolitis (NEC), have not been tested. The aim of this study was to evaluate if rhIGF-1/BP-3 supplementation in the first days after birth negatively affects clinical variables like growth, physical activity, blood chemistry and hematology and gut maturation (e.g., intestinal permeability, morphology, enzyme activities, cytokine levels, enterocyte proliferation, NEC lesions), using NEC-sensitive preterm pigs as a model for preterm infants.

**Methods:** Preterm pigs were given twice daily subcutaneous injections of rhIGF-1/BP-3 or vehicle. Blood was collected for IGF-1 measurements and gut tissue for NEC evaluation and biochemical analyses on day 5.

**Results:** Baseline circulating IGF-1 levels were low in preterm pigs compared with near-term pigs reared by their mother (<20 vs. 70 ng/ml). Injection with rhIGF-1/BP-3 resulted in increased plasma IGF-1 levels for up to 6 h after injection (>40 ng/mL). rhIGF-1/BP-3 treatment reduced the incidence of severe NEC lesions (7/24 vs.16/24, *p* = 0.01) and overall NEC severity (1.8 ± 0.2 vs. 2.6 ± 0.3, *p* < 0.05, with most lesions occurring in colon). In the small intestine, villi length (405 ± 25 vs. 345 ± 33 μm) and activities of the brush border peptidases aminopeptidase N and dipeptidylpeptidase IV were increased in rhIGF-1/BP-3 treated pigs, relative to control pigs (+31–44%, both *p* < 0.05). The treatment had no effects on body weight, blood chemistry or hematology, except for an increase in blood leucocyte and neutrophil counts (*p* < 0.05, i.e., reduced neonatal neutropenia). Likewise, rhIGF-1/BP-3 treatment did not affect intestinal tissue cytokine levels (IL-1β, IL-6, IL-8, TNFα,), enterocyte proliferation, goblet cell density, permeability or bacterial translocation to the bone marrow.

**Conclusion:** Supplemental rhIGF-1/BP-3 did not negatively affect any of the measured variables of clinical status or gut maturation in preterm pigs. Longer-term safety and efficacy of exogenous rhIGF-1/BP-3 to support maturation of the gut and other critical organs in preterm newborns remain to be investigated in both pigs and infants.

## Introduction

Preterm birth (<37 weeks gestation) accounts for 5–15% of all live births worldwide or about 15 million infants per year (1). Premature infants are a highly vulnerable population due to immaturity of their organs and the accelerated transition to extrauterine life. Postnatally, infants with gestational age (GA) <32 weeks at birth show clearly reduced serum levels of insulin-like growth factor 1 (IGF-1) relative to term infants and gestational age-matched fetuses *in utero*, probably due to the sudden loss of placental and amniotic fluid supplies (2–4). Recently, a large multi-center international clinical trial was initiated to test the clinical effects of supplemental recombinant human (rh) IGF-1/rhIGFBP-3 (rhIGF-1/BP-3) during the first weeks after extremely preterm birth (<28 weeks GA), with respiratory function as the primary outcome (ClinicalTrials.gov registry NCT03253263).

IGF-1 is a 70-amino acid polypeptide playing an important role in intrauterine and postnatal growth and organ development. In the fetus and newborn, the majority of circulating IGF-1 is generated by the liver in an insulin- and nutrient-dependent manner (4). About 80% of circulating IGF-1 is bound to IGF-binding protein (IGFBP)-3 and an acid labile subunit, regulating the action and biological availability of IGF-1 in peripheral tissues (5). Free IGF-1 binds to the type 1 IGF-receptor, a transmembrane tyrosine kinase receptor highly homologous to the insulin receptor, that activates intracellular signaling cascades like the mitogen-activated protein (MAP)-kinase and phosphatidylinositol 3(PI3-K)-kinase/Akt pathways (6, 7). In addition to its endocrine effects *via* the blood stream, IGF-1 is also produced locally in a variety of tissues exerting cellular paracrine and autocrine effects. In the gastrointestinal tract, IGF-1 may promote enterocyte proliferation and differentiation and inhibits apoptosis, thus supporting cell survival and mucosal growth (8–10). In young rodents, parenteral IGF-1 administration attenuates intestinal injury by decreasing apoptosis and increasing proliferation of epithelial cells after hypoxia/reoxygenation (11), sepsis (9) or thermally-induced damage (10). In weaned rats, an anabolic effect on the small intestine was observed only after subcutaneous, but not enteral IGF-1 administration (12). Inconsistent effects on intestinal growth have also been observed in neonatal pigs after enteral IGF-1 administration (13–15) and effects beyond the gut are poorly investigated. Considering the multifunctional actions of IGF-1, it is critical to understand how circulating IGF-1 levels relate to development of both the gut and other organs in the short and longer term.

Hellström et al. (16) demonstrated an association between persistently low serum concentrations of IGF-1 and several morbidities in preterm infants. In support, a recent phase II clinical trial indicated that exogenous rhIGF-1/BP-3 reduced the occurrence of severe bronchopulmonary dysplasia (BPD) and intraventricular hemorrhage (IVH) in extremely premature infants (17). The same study showed no effect on the overall incidence of necrotizing enterocolitis (NEC) (6/61 vs. 5/60), but the number of fatal NEC cases was highest in the rhIGF-1/BP-3 group (3 vs. 0). Generally, NEC is associated with high mortality (20–30%) and occurs in 5–10% of extremely preterm infants within the first weeks of life (18–22). Despite comprehensive research, in part using animal models, the pathogenesis of NEC remains ambiguous and worldwide the NEC incidence is constant or increasing (19, 21).

Preterm pigs reflect many clinical aspects of preterm infants, thus enabling pathophysiological investigation and drug development. Comparison between preterm pigs and preterm infants is organ-dependent but judged by their immature gut and immune systems, and high sensitivity to NEC and sepsis, 90% gestation in pigs may reflect gut and immunity development in extremely preterm infants (23–25). Within a few days of advancing enteral feeding with infant formula, 40–70% of preterm pigs spontaneously develop NEC-like lesions, including pneumatosis intestinalis, mucosal necrosis, villus atrophy and digestive dysfunction (26–29). We have previously shown that circulating IGF-1 levels remain low until at least 26 days after birth in preterm pigs vs. term pigs (30). However, data concerning the relationship between IGF-1 levels and comorbidities in preterm pigs are lacking.

We hypothesized that parenteral rhIGF-1/BP-3 supplementation in preterm pigs, to reach physiological levels in corresponding term individuals, would not negatively affect clinical variables, gut maturation and NEC incidence. To test this hypothesis, we first compared circulating IGF-1 levels in preterm pigs with values in near-term pigs reared artificially or reared naturally by their sow. Next, we tested the pharmacokinetics (PK) of rhIGF-1/BP-3 to establish an appropriate dosing regimen in preterm pigs. Finally, we investigated how restoration of circulating IGF-1 levels in preterm pigs affected clinical variables, parameters of gut function and NEC in 5-day-old formula-fed preterm pigs, known to be highly sensitive to spontaneous development of NEC-like lesions throughout the gut.

## Materials and Methods

Animal studies were conducted in accordance with the European Communities Council Directive 2010/63/EU for the protection of animals used for experimental purposes and approved by the Danish Animal Experiments Inspectorate, Ministry of Environment and Food of Denmark.

### Experiment 1: Basal Circulating IGF-1 Levels in Piglets

**Figure 1:**
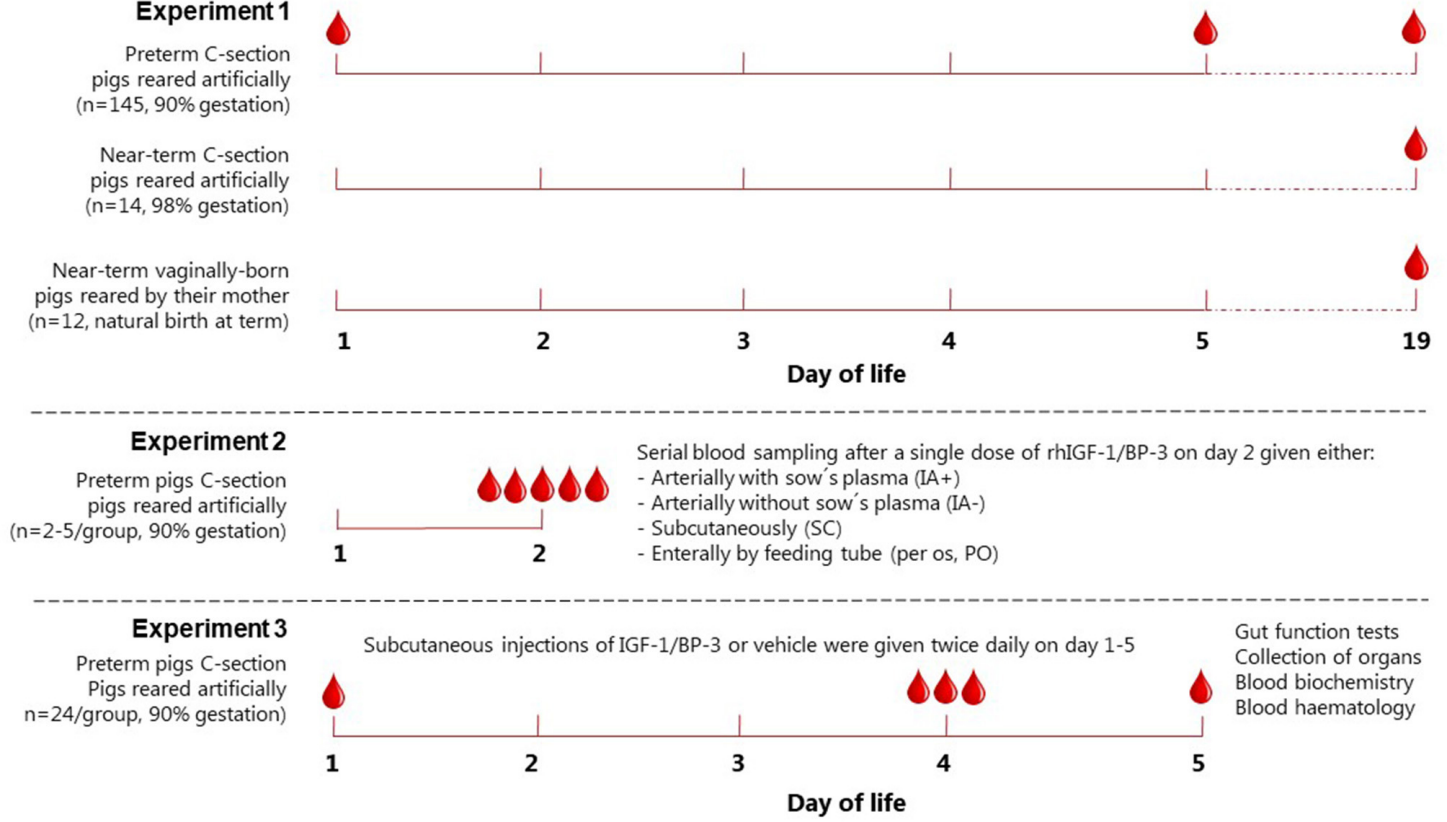
Experimental setup. Schematic drawing showing the experimental setup used to evaluate basal IGF-1 levels in piglets (Experiment 1), pharmacokinetics of IGF-1(Experiment 2) and the effects of rhIGF-1/BP-3 supplementation (Experiment 3). IA+ and IA-, intra-arterial administration with (+) or without (–) maternal plasma immunization; SC, subcutaneous; PO, peroral.

Groups of pigs (Landrace x Yorkshire x Duroc) were born at different gestational ages in relation to normal full term [117 ± 2 days] and reared artificially or naturally, as previously described (31). In brief, these pigs were born vaginally on day 112 and housed with their mother under natural rearing conditions (*n* = 12), by cesarean section at day 115 and housed in incubators (*n* = 14), or groups of pigs delivered by cesarean section on day 106 and housed in incubators (total *n* = 145). Blood samples were obtained at day 1 (cord blood), day 5 or day 19 (Figure 1).

### Experiment 2: Pharmacokinetics of IGF-1

A 1:1 molar ratio of the non-covalent complex rhIGF-1/rhIGFBP-3 (rhIGF-1/BP-3, mecasermin rinfabate) and formulation vehicle were provided by Takeda, Cambridge, MA, USA. Based on our literature studies from both infants and pigs (3, 4, 30), we aimed for a target physiological IGF-1 range of 30–110 ng/ml in the circulation. Preterm pigs for the pharmacokinetic study were delivered from one sow by cesarean section at day 106 and reared in incubators. All pigs received 4 ml/kg/h total parenteral nutrition (TPN) for 3 days. On day 2, pigs were divided into four groups, with pigs given a single injection of rhIGF-1/BP-3 (Figure 1): Pigs injected with 0.5 mg/kg intra-arterially without maternal plasma (IA-, *n* = 3), pigs injected with 0.5 mg/kg intra-arterially subsequent to 16 ml/kg maternal sow plasma (IA+, *n* = 3), pigs injected with 0.5 mg/kg subcutaneously with no sow's plasma (n=5) and pigs injected with 6 mg/kg orally, no maternal sow plasma and fed 10 ml/kg colostrum 0 and 1 h after IGF-1 (*n* = 2). Plasma for IGF-1 measurement was obtained *via* the umbilical arterial catheter at intervals from 30 min to 8 h after the single rhIGF-1/BP-3 injections. The preliminary investigation of IGF-1 levels after oral administration was to verify that the contribution from dietary IGF-1 to circulating IGF-1 levels would be minimal. Likewise, the studies with/without sow's plasma were to study the specific contribution of IGF-1 from sow's plasma, normally given in our studies on preterm pigs to provide them with passive immunity for survival and infection resistance (31). Based on the pharmacokinetic profile, with sustained levels of plasma IGF-1 within the desired physiological range (Supplementary Figure 1), a subcutaneous dose of 0.5 mg/kg rhIGF-1/BP-3 twice daily was chosen for the IGF-1 supplementation study (Experiment 3).

### Experiment 3: IGF-1 Supplementation Study

Forty-nine piglets were delivered from two sows by cesarean section at 90% of gestation (day 106). Immediately after birth all pigs were injected intramuscularly with 0.1 ml doxapram and 0.1 ml flumazenil, transferred to our NICU facility and housed in temperature-regulated incubators with extra oxygen supply (1–2 l/min) for the first 12 h. The pigs were fitted with orogastric and umbilical arterial catheters for enteral feeding and vascular access. One pig was euthanized due to catheter-related problems and excluded from the study before randomization. Pigs were block-randomized according to birth weight and gender into two groups: pigs given 0.5 mg/kg rhIGF-1/BP-3 subcutaneously (SC) twice daily (rhIGF-1/BP-3, *n* = 24) at 7 am and 3 pm and pigs given equivalent volumes of vehicle SC twice daily (Controls, *n* = 24) at 7 am and 3 pm until they were euthanized at day 5 (Figure 1). *Via* the umbilical arterial catheter all pigs were immunized with maternal sow plasma (16 ml/kg) during the first 24 h and parenteral nutrition (PN) (Kabiven modified with Vamin, Soluvit, Vitalipid and Peditrace, all Fresenius Kabi, Uppsala, Sweden) was infused continuously throughout the study period (2–5 ml/kg/h) to meet nutrient and fluid requirements of pigs (25). Every 3 h, the pigs were fed enteral formula composed of commercially available products used for feeding infants (formula composition shown in Supplementary Table 1). The enteral nutrition (EN) was provided *via* the orogastric catheter with increasing volumes (24–120 ml/kg/d) throughout the study period.

### Clinical Observations and *in vivo* Tests

Pigs were weighed daily, monitored closely and euthanized immediately if severe respiratory distress or NEC symptoms were observed. Clinical and fecal status were evaluated twice daily using previously validated scoring systems (30) (except that fecal score 5 included diarrhea with visual blood). All involved personnel was trained and experienced in work with preterm pigs. The treatment groups were blinded by color codes on the stable records. Motor activity was captured by infrared video surveillance connected to a motion detection software (PigLWin, Ellegaard Systems, Faaborg, Denmark). Physical activity was analyzed from day 2, 8 am, since physical activity during the first 24 h of life mainly reflects wake-up movements after delivery and anesthesia. Intestinal permeability was assessed day 5 by peroral administration of a 5/5% lactulose/mannitol bolus (15 ml/kg) and urine recovery after 3 h. Concentration of lactulose and mannitol in urine were obtained and analyzed as previously described (32). Gastric residual was assessed by oral administration of a last feeding bolus (15 ml/kg) 60 min prior to euthanasia and postmortem measurement of the stomach content weight. Gastric pH was measured in the stomach content using a pH meter.

### Blood Biochemistry, Hematology and Circulating IGF-1 Levels

For biochemistry and hematology, 1 ml blood was collected on day 4 in a heparin blood vacutainer (BD Diagnostics, Oxford, UK) *via* the umbilical catheter. Biochemistry of plasma and cell counting of full blood were analyzed using an Advia 1800 Chemistry System and an Advia 2120i Hematology system (Siemens Healthcare, Ballerup, Denmark), respectively. The plasma biochemistry profile included typical measures of metabolic, liver and kidney functions.

For circulating IGF-1 measurement blood samples were obtained two, six and 15 h post-dosing at day 4 *via* the umbilical catheter and at day 5 *via* intracardial puncture at euthanasia. One ml blood was collected in a serum tube with clot activator (BD diagnostics), rested until clotted (½-1 h), centrifuged at 1300 × *g* for 10 min and stored at −80°C. All serum samples were analyzed using a human IGF-1 ELISA kit (Mediagnost GmbH, Reutlingen, Germany) in a bioanalytical testing laboratory (PPD Laboratories, Richmond, Virginia). The quantitative limit of the analysis was 20 ng/mL, however, values between 10 and 20 ng/ml were also recorded for information. The detection limit of the analysis was 10 ng/ml and measurements lower than 10 ng/ml were assigned a value of 5 ng/ml for quantitative evaluations.

### Tissue Collection and NEC Evaluation

On day 5, pigs were euthanized with an intracardial injection of sodium-pentobarbital (Euthanimal, Scanvet, Denmark). The entire gastrointestinal tract was removed from the abdomen and the stomach, proximal, middle and distal small intestine, caecum and colon were evaluated for pathological changes in a blinded fashion by two persons, using the following validated NEC scoring system for each of the gut regions (28): 1 = absence of lesions, 2 = local hyperaemia, 3 = hyperaemia, extensive edema and local hemorrhage, 4 = extensive hemorrhage, 5 = local necrosis or pneumatosis intestinalis, 6 = extensive necrosis and pneumatosis intestinalis. NEC was defined as a score of minimum 3 in at least one gut region, whereas a score of minimum 4 in at least one gut region was defined as “severe NEC.” An average NEC severity score of the total gastrointestinal tract was calculated as the mean of the highest score of the small intestine parts (prox, mid or dist), the highest score of the colon parts (cecum or colon) and stomach. Organs were weighed and tissue from small intestine and colon was snap frozen and stored at −80°C to evaluate enzyme activities and levels of pro-inflammatory cytokines. Bone marrow was harvested from the distal femoral epiphysis with sterile instruments, cultured on blood agar at room temperature for 24 h, and stored at 4°C until colonies were enumerated.

### Histological Examinations

Mid small intestine and colon tissue were fixed in 4% formaldehyde solution (CellPath, Newton, Powys, United Kingdom) routinely processed, embedded in paraffin blocks and sectioned at a thickness of 3 μm. The slides were stained with hematoxylin and eosin and Alcian-Blue-periodic acid Schiff for light-microscopic evaluation. Using a Leica DM2500 optical microscope images were taken by a lab technician unaware of treatment groups and villus length and crypt depth were quantified by an averaging value from 10 representative villi and crypts measured by ImageJ software (Laboratory for Optical and Computational Instrumentation, University of Wisconsin-Madison). Goblet cell density was calculated by the same lab technician using a stereological approach as the area of goblet cells relative to the total area of tunica mucosa (%).

To access the degree of proliferation, immunohistochemical staining was performed on tissue from small intestine mid sections. The slides were pre-treated with TEG buffer pH 8 for 2x5 min. Endogenous peroxidase was inhibited by incubation in 0.6% H_2_O_2_ for 15 min followed by Ultra V blocking (Thermo Fisher Scientific, Hvidovre, Denmark) for 5 min. Primary Ki-67 antibody (clone MIB-1, Dako, Glostrup, Denmark) was diluted 1:200 in 1% bovine serum albumin (BSA)/Tris-buffered saline (TBS) and slides were stained with the primary antibody overnight. Afterwards the sections were incubated for 20 min with Primary Antibody Enhancer followed by detection with HRP Polymer for 30 min (Thermo Fisher Scientific). Slides were then incubated with aminoethyl carbazole (AEC) solution for 10 min and counterstained with Meyer's hematoxylin. Finally, sections were dehydrated and mounted with glycerol-gelatine. Using a Leica DM2500 optical microscope, eight images of each small intestine section were taken in a standardized way and an automated digital image analysis of positive Ki67 staining areas in tunica mucosa was performed using FIJI software (Fiji Is Just ImageJ, Laboratory for Optical and Computational Instrumentation). The analysis was run with vectors H DAB to detect cell nuclei and staining of specific Ki76 immunohistochemically positive areas (threshold = ∞-150, particle size, pixel^∧^2 = 10-∞). The process was followed by a calculation step. Ki67 density was given as mean total Ki67 stained area of the total nuclei area in tunica mucosa (%).

### Brush Border Enzyme Activity and Cytokine Expression

For activity of the brush border enzymes, lactase, maltase, sucrase, aminopeptidase N (ApN), aminopeptidase A (ApA), dipeptidylpeptidase IV (DPPIV) tissue samples from the mid small intestine were homogenized in 1% Triton X-100 buffer using a gentleMACS Dissociator (Miltenyi Biotec, Bergisch Gladbach, Germany). Assays were performed using specific substrates, as described previously (33). Interleukin (IL)-1β, IL-6, IL-8 and tumor necrosis factor (TNF)-α levels in mid small intestine and colon homogenates were analyzed using DuoSet ELISA kits (R&D systems, Minneapolis, MN) following the manufacturer's instruction. Each sample was tested in duplicate and measurements below detection limits were assigned a value of zero for the given analyte.

### Statistical Analysis

The statistical software package R (version 3.6.1) was used for statistical analyses. Statistical significance of difference in continuous variables was analyzed by linear models if normally distributed. The model was tested for normality by Shapiro-Wilk's test and plots, data were transformed when required and non-parametric analysis was applied when data could not be transformed properly. Basal plasma IGF-1 levels measured in in Experiment 1 were analyzed by a non-parametric test with corrections for multiple testing. Binary data were analyzed by a logistic regression model. Ordered categorical outcomes, like NEC scores, were analyzed by a proportional odds logistic regression model. Repeated measurements were analyzed by the linear mixed effects modelif continuous and by cumulative link mixed models if ordered categorical. The above mentioned models were adjusted for possible cofounders like litter, sex and birth weight. For value comparisons, *p* < 0.05 was considered statistically significant. Values are expressed as mean ± standard error of mean (SEM) unless otherwise specified and levels of significance are assigned as *p < 0.05, **p < 0.01, ***p < 0.001.

## Results

### Experiment 1: Basal IGF-1 Levels in Piglets

**Figure 2:**
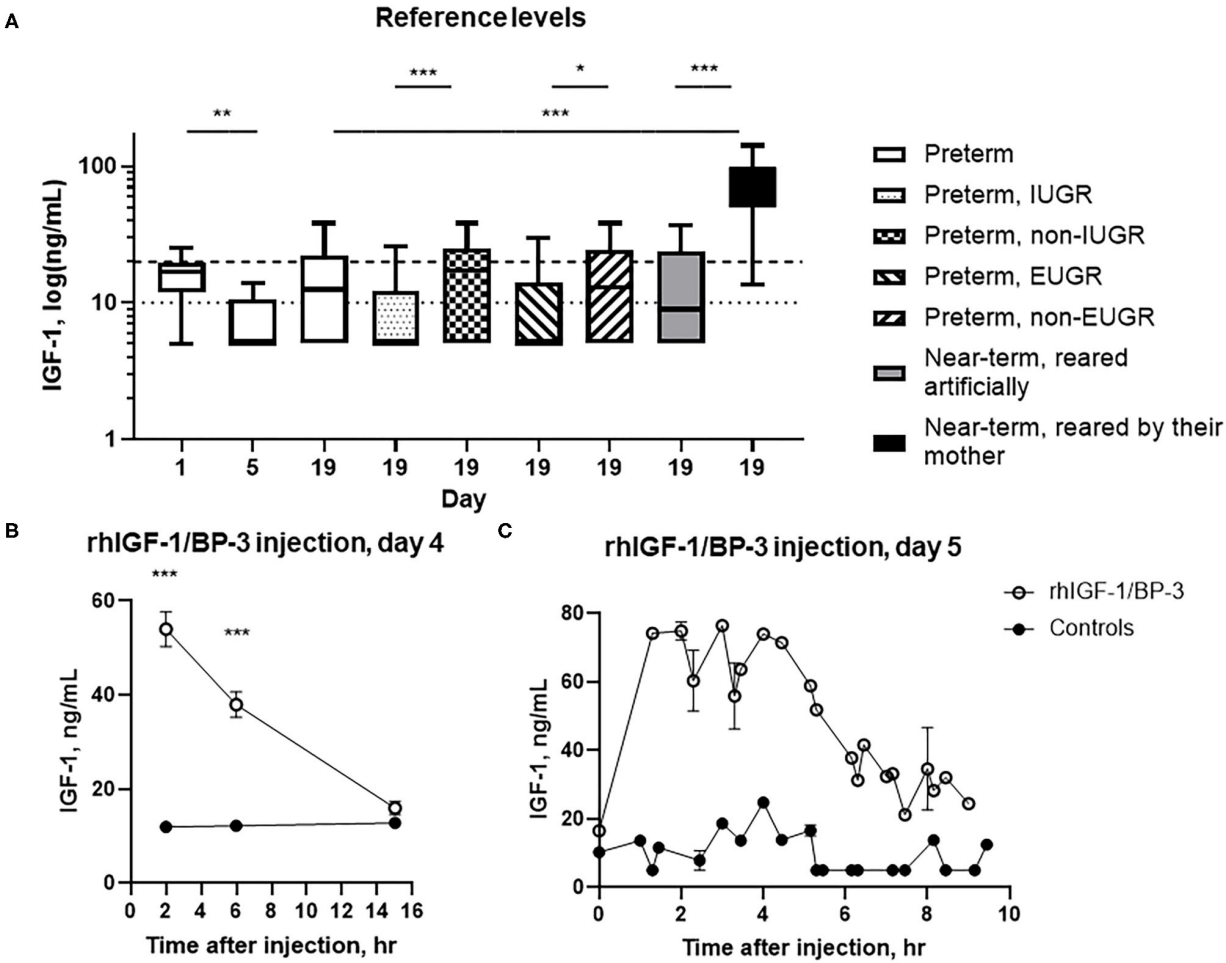
Circulating IGF-1 levels and pharmacokinetics (mean ± SEM). **(A)** Basal IGF-1 plasma levels in 1–19 day-old preterm pigs reared in incubators (*n* = 7–107), near-term pigs reared in incubators (*n* = 14), and near-term pigs reared with their mother (*n* = 12). The 19 day-old preterm pigs were subdivided into pigs with intrauterine growth restriction (IUGR, *n* = 28) or non-IUGR (*n* = 79), and extrauterine growth restriction (EUGR, *n* = 26) or non-EUGR (*n* = 80), with growth restriction defined as the pigs with body weight within the lowest 25% of the growth percentiles. Data are presented as box plots (median and 25/75 percentiles) with whiskers = min to max. The interrupted horizontal line indicates the lower limit of quantitation (20 ng/ml) and the dotted horizontal line indicates the limit of detection (10 ng/ml) of the assay. **(B)** Serum IGF-1 levels in rhIGF-1/BP-3 and control preterm pigs after a single subcutaneous injection of rhIGF-1/BP-3 or vehicle on day 4 (*n* = 18–23) (mean ± SEM). **(C)** Serum IGF-1 levels at euthanasia 0–10 h after the last subcutaneous injections of rhIGF-1/BP-3 or vehicle on day 5 (*n* = 1–2 for samples taken 1-10 h after last dosing). *t* = 0 represents samples taken 15 h after last dosing (*n* = 16–19). *p < 0.05, **p < 0.01, ***p < 0.001.

At day 1, 5, and 19, 23% (7/31), 0% (0/5) and 29% (31/107) of preterm pigs had plasma IGF-1 values above the pre-set lower quantitative limit of the assay, with an estimated mean level below 20 ng/ml. At day 19, more near-term pigs reared by their sow had IGF-1 values above the quantitative limit (83%, 10/12) and a higher estimated mean level than preterm pigs (both *p* < 0.01) and near-term pigs reared artificially (36%, 5/14 above the quantitative limit, both *p* < 0.05) (Figure 2A). Preterm pigs born with low birth weight [lowest 25% weight percentile (28/107)] showed a reduced estimated mean level at day 19 compared with remaining preterm pigs (*p* < 0.001, Figure 2A). Also, preterm pigs with extrauterine growth restriction (lowest 25% weight gain percentile, 26/106) had reduced estimated mean levels compared with the remaining pigs (*p* < 0.05, Figure 2A).

### Experiment 2: Pharmacokinetics of IGF-1

Baseline plasma IGF-1 levels (*t* = 0) in preterm pigs were generally below the lower limit of quantitation, with an estimated mean level below 20 ng/ml. Following intra-arterial dosing of 0.5 mg rhIGF-1/BP-3, plasma IGF-1 levels peaked 30 min after injection and reached baseline levels by 8 h both in the IA+ and IA- group (Supplementary Figure 1, i.e., passive immunization with sow's plasma contributed minimally to IGF-1 levels). Following subcutaneous administration, IGF-1 levels peaked at 4 h and had sustained elevated levels within the desired physiological range (30–110 ng/ml) for up to 8 h. Finally, following oral administration of 6 mg/kg rhIGF-1/BP-3, plasma IGF-1 levels were comparable to baseline levels at all time points (Supplementary Figure 1, i.e., minimal enteral absorption of IGF-1).

### Experiment 3: IGF-1 Supplementation Study

#### Circulating IGF-1 Levels

At birth, serum IGF-1 levels in cord blood of rhIGF-1/BP-3 and controls pigs were similar and generally near the quantitative limit of 20 ng/ml (*n* = 24–25). Two, six and 15 h after injection on day 4, the serum IGF-1 levels in pigs treated with rhIGF-1/BP-3 were 54.0 ± 3.6, 38.0 ± 2.6 and below 20 ng/ml, respectively (mean ± SEM). In corresponding control pigs, the estimated IGF-1 levels were below 20 ng/ml throughout (Figure 2B). When pigs were euthanized in a random sequence on day 5, serum IGF-1 levels in rhIGF-1/BP-3 pigs peaked 1.5-4.5 hrs after the last injection, with IGF-1 levels being 55–75 ng/ml. The IGF-1 levels then declined to 20–35 ng/ml 7–9 h after injection (Figure 2C). In all control pigs, values were below 20 ng/ml (Figure 2C, except for one control pig with a value of 24.8 ng/ml).

### Clinical Observations, Organ Weights, Blood Chemistry, and NEC Lesions

Until day 4 all pigs were clinically stable with a clinical score = 1 (Supplementary Table 2). One control pig was euthanized on day 4 because of severe clinical NEC symptoms (abdominal distension, cyanosis and bloody diarrhea) while all others were killed on day 5 for planned tissue collection. No differences were found between rhIGF-1/BP-3 pigs and control pigs in birth weight, kill weight and average daily weight gain (Table 1). Likewise, rectal temperatures, clinical scores, fecal scores and physical activity were similar throughout the study period (Supplementary Table 2). At tissue collection on day 5, there were no differences among groups in relative organ weights, intestinal length or gastric pH (Table 1). At this time, hematological values and blood biochemistry values were similar between groups, except that rhIGF-1/BP-3 treatment increased the leukocyte and neutrophil counts (*p* < 0.05, Supplementary Tables 3, 4). Of note, basal plasma glucose levels were not significantly affected by rhIGF-1/BP-3 treatment.

**Table 1:** Relative organ weights and parameters of preterm pigs treated with rhIGF-1/BP-3^*^.

**Parameter**	**rhIGF-1/BP-3**	**Controls**
Number of animals	23–24	22–24
Birth weight, g	1164 ± 63	1180 ± 60
Kill weight, g	1232 ± 66	1249 ± 61
Daily weight gain, g	14.9 ± 2.7	15.3 ± 2.5
SI length, cm/kg	285.7± 9.9	280.4 ± 8.0
SI weight:length, mg/cm	91.8 ± 2.6	89.9 ± 3.2
Stomach acidity, pH	3.8 ± 0.1	3.8 ± 0.1
**Relative organ weights, g/kg:**		
Liver	27.4 ± 0.7	28.0 ± 0.6
Spleen	2.7 ± 0.2	2.4 ± 0.1
Kidney	8.7 ± 0.3	8.2 ± 0.3
Stomach	6.2 ± 0.2	6.8 ± 0.4
Stomach content	25.8 ± 1.6	27.4 ± 2.5
Colon	16.0 ± 0.6	16.3 ± 0.7
SI total	25.8 ± 0.6	24.9 ± 0.7
SI Proximal	9.1 ± 0.3	8.6 ± 0.4
SI Middle	7.9 ± 0.6	7.3 ± 0.4
SI Distal	7.7 ± 0.2	7.6 ± 0.2

* *Values are mean ± SEM. There were no significant differences for any variable between groups (all p > 0.05). SI, Small intestine*.

**Figure 3:**
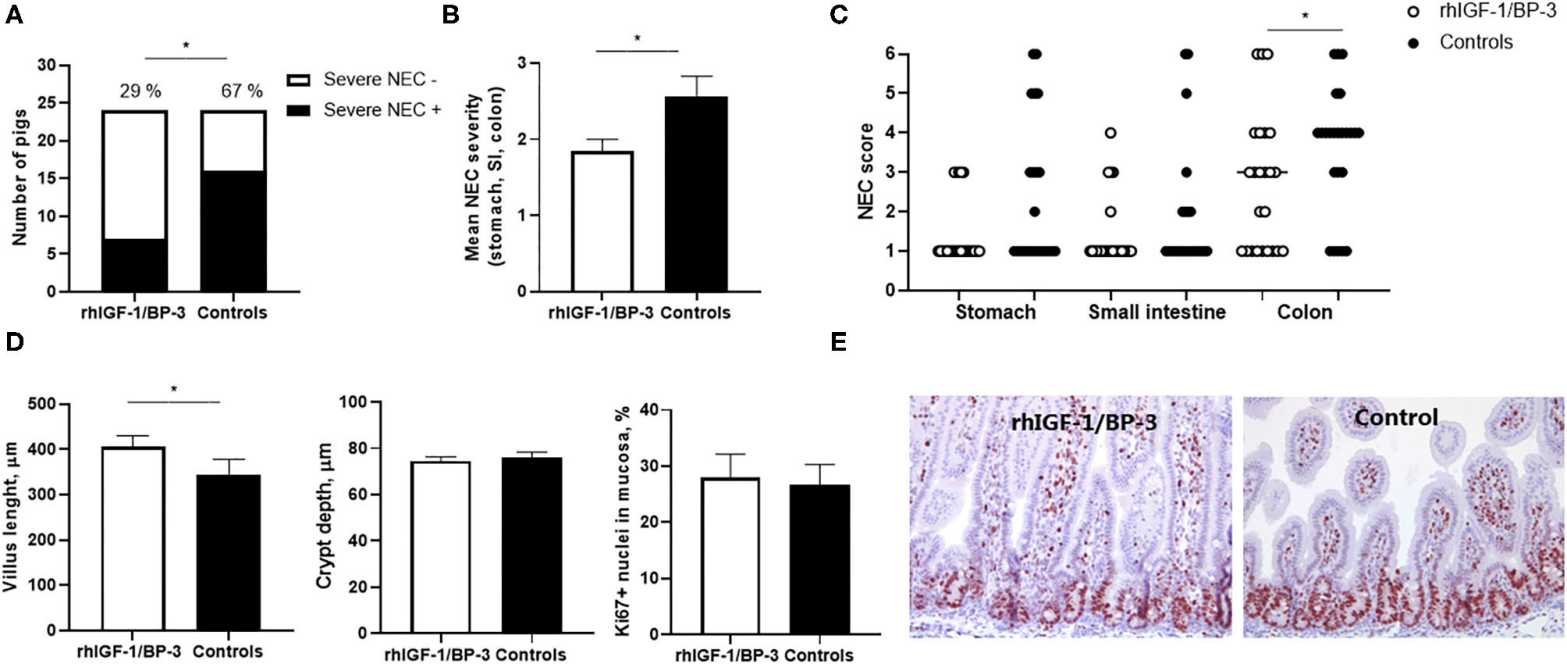
Intestinal lesions and structure. **(A)** Incidence of severe NEC (defined as score ≥4 in at least one gut region) in rhIGF-1/BP-3 and control preterm pigs (*n* = 24). **(B)** NEC severity score across gut regions in rhIGF-1/BP-3 and control preterm pigs (*n* = 24). **(C)** Individual NEC scores in stomach, small intestine regions (highest score of the proximal, middle and distal section) and colon regions (highest score of the colon and cecum). **(D)** Villus length, crypt depth and Ki67 stained area relative to total nuclei area in tunica mucosa in mid small intestine (*n* = 23). Values are mean±SEM. **(E)** Immunohistochemically staining for Ki67 with hematoxylin counterstain in a representative middle small intestine section from a preterm pig treated with rhIGF-1/BP-3 and a control preterm pig. Pictures are 10x. *p < 0.05, **p < 0.01, ***p < 0.001.

When the incidence of NEC lesions included mild cases (score ≥3 in at least one gut region) there was no significant difference between groups (15/24 in rhIGF-1/BP-3 pigs vs. 20/24 in control pigs), but severe NEC lesions (score ≥4 in at least one gut region) was less frequent in rhIGF-1/BP-3 pigs than in control pigs (7/24 vs.16/24, *p* = 0.01, Figure 3A). Accordingly, average NEC severity across the entire gut and NEC scores in the colon region were lower in rhIGF-1/BP-3 vs. control pigs (Figures 3B,C, both *p* < 0.05). Stomach, small intestine and colon NEC lesions were present in 13 (37%), 8 (23%), and 32 (91%) out of the total 35 NEC cases, respectively (Supplementary Table 5). Thus, colon lesions occurred most frequently in 5 day-old preterm pigs, with three pigs (9%) having NEC in both colon and small intestine, nine pigs (26%) having NEC in both colon and stomach and 3 pigs (9%) having NEC in all three gut regions (Supplementary Table 5).

### Intestinal Histology, Permeability, Digestive Enzymes and Cytokines

In the mid small intestine, rhIGF-1/BP-3 pigs had increased villus length compared with control pigs (*p* < 0.05, Figure 3D). When the four pigs with severe NEC lesions in this region (lesion score ≥ 4) were excluded from the analysis, the villus height difference between groups disappeared, indicating that the rhIGF-1/BP-3 effect on villus morphology occurred *via* a NEC-protective effect. No differences in crypt depth or Ki67 density, reflecting the enterocyte proliferation rate, were observed (Figures 3D,E). In the colon, crypt depth (63.9 ± 1.6 vs. 64.1 ± 2.5 μm) and density of mucin-containing goblet cells (5.9 ± 0.7 vs. 5.3 ± 0.9 %) did not differ between rhIGF-1/BP-3 and control pigs.

**Figure 4:**
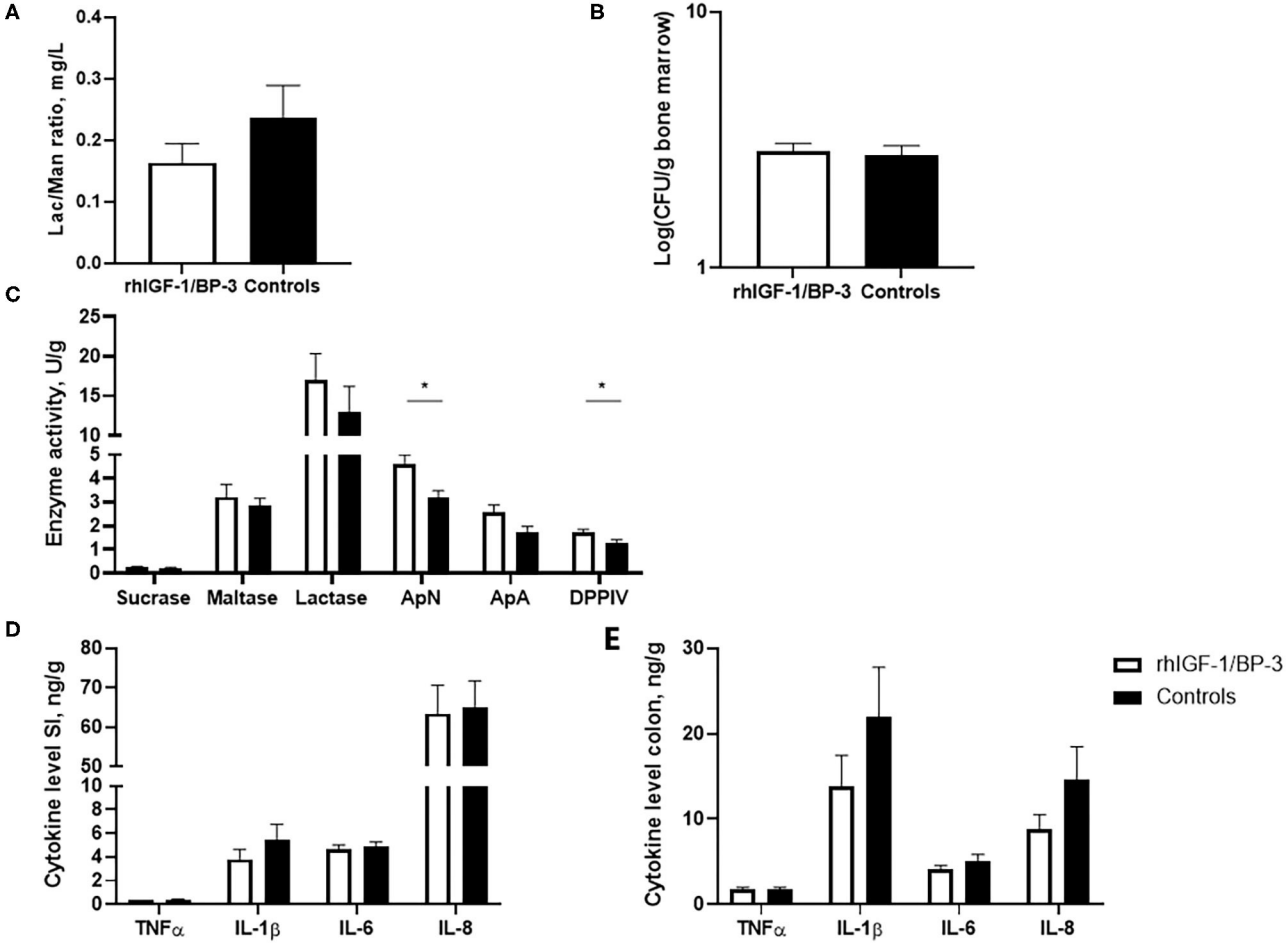
Intestinal functions (means ± SEM). **(A)** Concentration of lactulose/mannitol (Lac/Man) ratio to asses intestinal permeability in the urine on day 5 (*n* = 18–22), and **(B)** bacterial load in bone marrow (colony forming units, CFU) to assess bacterial translocation on day 5 (*n* = 23–24). **(C)** Brush border enzyme activities in mid small intestine (*n* = 24, ApN, aminopeptidase N; ApA, aminopeptidase A; DPPIV, dipeptidylpeptidase). Pro-inflammatory cytokines in **(D)** middle small intestine and **(E)** colon (*n* = 23–24). *p < 0.05, **p < 0.01, ***p < 0.001.

No significant differences were observed between rhIGF-1/BP-3 and control pigs in intestinal permeability, as indicated by the urinary lactulose/mannitol ratio and accumulation of bacteria in the bone marrow (Figures 4A,B). Relative to control pigs, rhIGF-1/BP-3 pigs had a 44% (*p* = 0.01) and 31% (*p* = 0.02) increase in activity of the brush border peptidases ApN and DPPIV respectively, while ApA and disaccharidase activities (sucrase, maltase, lactase) did not differ between groups (Figure 4C). No significant differences in TNFα, IL-1β, IL-6 or IL-8 levels in the mid small intestine or colon were observed (Figures 4D,E).

## Discussion

Considering the ongoing clinical trials on IGF-1 supplementation in extremely preterm infants, it is important to understand how elevated IGF-1 levels in the postnatal period may affect organ development in preterm neonates short- and long-term. Using preterm NEC-sensitive pigs as models for such infants, we now examined the short-term effect of supplemental rhIGF-1/BP-3 on clinical variables, gut maturation and NEC incidence. Supporting our earlier studies (30), circulating IGF-1 levels remained low in preterm pigs for at least 3 weeks after birth (until the weaning transition period), at levels similar to those reported in hospitalized very and extremely preterm infants (2, 4). Conversely, IGF-1 values in 3 week-old near-term pigs reared by their sow were similar to values for normal *in utero* human fetuses in the weeks before full-term birth (4). In control preterm pigs, we could only estimate the basal IGF-1 levels as most of the values were below the limit of optimal quantitation for our assay. Thus, a window of opportunity for supplemental IGF-1 may exist in the first week(s) after preterm birth. Our results suggest that IGF-1 supplementation in the first days after birth, restoring circulating IGF-1 levels for up to 6 h twice daily for 4 days, did not negatively affect clinical parameters, gut function or NEC. Rather, the treatment induced a moderate reduction in NEC incidence, accompanied by increased intestinal villus heights and peptidase activities that are good markers of villus integrity and function in preterm pigs (25–29). Further studies in both pigs and infants are required to confirm these findings.

It is well-documented that IGF-1 stimulates enterocyte proliferation and function (10, 12, 14, 15) but we were unable to demonstrate effects on epithelial proliferation, as tested by immunohistochemical staining. Enterocyte proliferation is relatively slow in fetuses and preterm neonates, especially in the more distal parts of the intestine, and IGF-1 may have worked to increase enterocyte lifespan and differentiation rather than stimulating proliferation, like in rodents after intestinal injury (9–11) or preterm pigs given milk diets rich in IGF-1 (34), supporting the effects of oral IGF-1 on gut growth, nutrient absorption, and enzyme activities in term pigs (13–15). Fresh milk contains important nutrients and multiple bioactive factors and the beneficial effects of mothers' milk and colostrum on gut maturation and NEC in infants are well-documented (20, 35). Colostrum and milk feeding also improve gut function and reduce NEC incidence and severity in preterm pigs relative to formula feeding (27, 29, 36). This may in part relate to high IGF-1 exposure (37, 38). Industrial processing of bovine milk for infant formula, for example, filtration and heat treatment steps, reduces the concentrations of bioactive proteins, including IGF-1 (34).

Impaired innate and adaptive immunity is a hallmark of prematurity and IGF-1 may play a role in this. In both preterm pigs and infants, a low number of leucocytes, specifically neutrophils (neonatal neutropenia), may contribute to high susceptibility to infection, sepsis and NEC (23, 39, 40). However, in this study no correlation was observed between the number of leucocytes in the blood and the number of bacteria in the bone marrow of individual pigs (data not shown). In infants, IGF-1 has anti-inflammatory effects on newborn cord blood (41) and in rodents IGF-1 inhibits the TLR4/NF-κB signaling pathway and secretion of pro-inflammatory cytokines (42). The neutropenia and depressed immune function in preterm infants (40) and pigs (23, 39) may be partly prevented by IGF-1 treatment, like in other life stages (7, 43), but more studies are required to test this hypothesis.

Locally in intestinal epithelial cells, IGF-1 increases the expression of mucin and SIgA and protects against oxidative injury, thereby promoting mucosal barrier functions (8, 42). In this study, the macroscopic IGF-1 effects in the colon were not accompanied by changes in tissue cytokine levels or mucosal barrier function in the small intestine, as indicated by the urinary lactulose/mannitol ratio, bacterial load in the bone marrow or goblet cell density, despite that the overall NEC severity was similar to that reported in earlier similar studies in preterm pigs (39, 44, 45). However, some of these parameters may be mostly affected by NEC lesions in the small intestine vs. colon (where lesions occurred most frequently in this study), as shown in our previous study on region-dependent NEC effects on systemic inflammation in preterm pigs (46). In follow-up studies, we have quantified the expression levels of a series of immune-related genes specifically in the colon region (C3, CASP3, HIF1A, IL17, IL8, LBP, MPO, OFLM4, TLR2, TLR4, VEGF) but no significant differences were found between rhIGF-1/BP-3 pigs and control pigs on day 5 (unpublished observations). We conclude that the chosen treatment regimen, like dose, time and length of administration, had minimal effects on both intestinal and colonic immune responses in preterm pigs, despite the observed NEC-protective effects.

In the recent Swedish infant rhIGF-1/BP-3 intervention study, treatment reduced BPD and IVH severity, while the primary endpoint (retinopathy of prematurity, ROP) was unaffected (17). Conclusions regarding NEC effects were not possible due to low sample size and low NEC rate. The overall incidence of NEC was similar between groups (5–6 out of 60–61 infants), but the number of fatal NEC cases was highest in the rhIGF-1/BP-3 treated group (3 vs. 0), which raises some concerns despite the low number of cases. NEC remains a frequent and serious disease with potential fatal outcomes or long-term consequences for infants (18, 19, 21, 47). Current treatment strategies include bowel rest, nasogastric decompression, systemic antibiotics, respiratory and cardiovascular support and in the worst cases, surgical intervention (25–50% of NEC cases) (35, 48).

Preterm infants are a highly heterogeneous patient group and standardized rhIGF-1/BP-3 supplementation of all extremely preterm infants is unlikely to be appropriate. Because of the short circulating half-life of IGF-1 after subcutaneous and intravenous injections with rhIGF-1/BP-3, a current need for daily administration *via* indwelling catheters or repeated injections, is another challenge that needs to be overcome and adapted to clinical practice. The present pre-clinical safety and efficacy results in preterm pigs are promising but results should be interpreted with caution. Importantly, the short duration of twice daily subcutaneous dosing and the short half-life of rhIGF-1/BP-3 did not result in longer-lasting, continuous physiologic levels of circulating IGF-1 in preterm pigs. This may partly explain the limited effects and benefits across all the investigated clinical, blood and tissue parameters in preterm pigs during the first 5 days of life. We have recently tested the effects of a longer, higher and more frequent dose of rhIGF-1/BP-3 (0.75 mg/kg three times daily until day 9 after birth) and confirmed the reduction in NEC incidence, together with a clear increase in intestinal weight (unpublished observations). Provision of rhIGF-1/BP-3 at the right time and dose may be safe and support growth and organ development in subgroups of preterm infants, but more studies, across multiple organs, are required before rhIGF-1/BP-3 can used in clinical practice for groups of preterm infants.

Prematurity involves developmental deficits across multiple organs in the body, making exogenous IGF-1 an attractive early therapy for very preterm infants with its well-documented multi-functional physiological benefits in animal models and low levels after preterm birth in humans. Neonatal IGF-1 supplementation is clearly a therapy where the benefits vs. potential unforeseen treatment risks must be thoroughly investigated. Importantly, organ systems develop differently in various mammals relative to the normal time of birth, either preterm or term. This makes it a challenge to translate the effects of exogenous IGF-1 observed for one organ system at one time point in one species (e.g., pigs) to corresponding conditions in another species (e.g., infants). Preterm pigs are more sensitive to develop NEC lesions than very preterm infants and lesions often first appear in the colon region, unlike infants (39, 44, 48). However in both infants and pigs, the predisposing factors for NEC lesions are similar, for example, low gestational age, aggressive enteral (formula) feeding and adverse gut bacterial colonization (25–27, 29, 44). These interact with a number of other determinants, eventually leading to breakdown of the gut mucosal barrier and excessive mucosal inflammation (20, 35).

Future studies, potentially using pigs as models, should further study both short- and long-term NEC effects of IGF-1 and other critical morbidities known from very preterm infants, like BPD, IVH and ROP. In such studies, it will also be important to verify if IGF-1 supplementation in early life may alleviate the growth restriction and nutrient dysmetabolism often observed in very preterm infants.

## Data Availability Statement

The raw data supporting the conclusions of this article will be made available by the authors, without undue reservation.

## Ethics Statement

The animal study was reviewed and approved by National Committee on Animal Experimentation, Denmark.

## Author Contributions

KH conceived and designed the experiments, acquired, analyzed and interpreted data, and wrote the paper. PS conceived and designed the experiments, analyzed and interpreted data, helped to draft the paper, and took final responsibility for its contents. XG and TT conceived and designed the experiments and acquired and interpreted data. XP, RN, and TM acquired data. TG analyzed and interpreted data. GC and NB conceived and designed the experiments. All authors revised the manuscript critically for important intellectual content and approved the final version. All authors agree to be accountable for all aspects of the work in ensuring that questions related to the accuracy or integrity of any part of the work are appropriately investigated and resolved.

## Conflict of Interest

TG, NB, RN, and GC were employed at Takeda, MA at the time of study. These co-authors did not participate in study execution, data acquisition or in drafting the manuscript with its text, results presentation, tables and figures. The remaining authors declare that the research was conducted in the absence of any commercial or financial relationships that could be construed as a potential conflict of interest.
