# Supplementary material for: Supplemental Insulin-Like Growth Factor-1 and Necrotizing Enterocolitis in Preterm Pigs
Source: Front Pediatr. 2021 Feb 4;8:602047. doi: 10.3389/fped.2020.602047 (PMC7891102; doi:10.3389/fped.2020.602047)
Supplement: Supplementary file 1 [file Table_1.pdf]

Supplementary table S1. Enteral milk formula composition

**Product**	**Amount (g/L)**	**Supplier**
Calogen LCT, lipid emulsion	20	Nutricia, Allerød, Denmark
Liquigen MCT, lipid emulsion	75	Nutricia
Fantomalt	20	Nutricia
Phlexy-Vits, vitamin and mineral mix	2	Nutricia
Miprodan 40	15	Arla Foods Ingredients
