# Supplementary material for: Supplemental Insulin-Like Growth Factor-1 and Necrotizing Enterocolitis in Preterm Pigs
Source: Front Pediatr. 2021 Feb 4;8:602047. doi: 10.3389/fped.2020.602047 (PMC7891102; doi:10.3389/fped.2020.602047)
Supplement: Supplementary file 2 [file Table_2.pdf]

Supplementary table S2. Clinical parameters of preterm pigs treated with rhIGF-1/BP-3*

**Parameter**	**rhIGF-1/BP-3**	**Controls**
Number of animals	21-24	18-24
Rectal temperature, 1 and 24 hrs after birth:	38.0 ±0.1	37.8 ±0.1
	38.2 ±0.1	38.2 ±0.2
Clinical score, median [min; max], morning and evening:		
Day 1	1 [1; 1]	1 [1; 1]
Day 2	1 [1; 1]	1 [1; 1]
	1 [1; 1]	1 [1; 1]
Day 3	1 [1; 1]	1 [1; 1]
	1 [1; 1]	1 [1; 1]
Day 4	1 [1; 1]	1 [1; 1]
	1 [1; 2]	1 [1; 4]
Day 5	1 [1; 2]	1 [1; 4]
Fecal score, median [min; max], morning and evening:		
Day 1	1 [1; 1]	1 [1; 1]
Day 2	1 [1; 1]	1 [1; 1]
	1 [1; 2]	1 [1; 2]
Day 3	1 [1; 2]	1 [1; 2]
	1 [1; 1]	1 [1; 1]
Day 4	4 [1; 4]	4 [1; 5]
	4 [1; 4]	4 [1; 5]
Day 5	4 [1; 5]	4 [2; 5]

Physical activity, % activity per hour:		
Day 2, 8 am – 8 pm	23.7 ±1.2	22.1 ±1.6
Day 2, 8 pm – Day 3, 8 am	18.8 ±1.2	17.9±1.4
Day 3, 8 am – 8 pm	18.7 ±1.1	17.6 ±1.9
Day 3, 8 pm – Day 4, 8 am	18.1 ±1.2	17.9±1.4
Day 4, 8 am – 8 pm	18.2 ±1.2	19.5 ±1.4
Day 4, pm – Day 5, 8 am	16.3 ±1.4	16.0 ±1.4

*Unless stated otherwise, values are mean ± SEM. There were no statistically significant differences in the variables (all $p > 0.05$). The clinical assessment criteria included respiratory distress, color, lethargy, abdominal distension and skin changes, as previously described (30). Each pig was assigned a score between 1 (clinically stable) and 4 (very ill, euthanasia to be considered)
