# Supplementary material for: Supplemental Insulin-Like Growth Factor-1 and Necrotizing Enterocolitis in Preterm Pigs
Source: Front Pediatr. 2021 Feb 4;8:602047. doi: 10.3389/fped.2020.602047 (PMC7891102; doi:10.3389/fped.2020.602047)
Supplement: Supplementary file 3 [file Table_3.pdf]

Supplementary table S3. Hematology of preterm pigs treated with rhIGF-1/BP-3 or vehicle*

**Parameter**	**rhIGF-1/BP-3**	**Controls**
Number of animals	20	21
Total leukocytes, 10^9/L	2.4 ±0.2*	1.8 ±0.1
Total erythrocytes, 10^9/L	3.8 ±0.1	3.6 ±0.1
Thrombocytes, 10^9/L	179.2 ±11.3	172.4 ±13.4
Neutrophils, 10^9 /L	1.2 ±0.1*	0.8 ±0.1
Lymphocytes, 10^9/L	1.1 ±0.1	0.9 ±0.1
Monocytes, 10^9/L	0.04 ±0.01	0.03 ±0.003
Eosinophils, 10^9/L	0.04 ±0.01	0.04 ±0.01
Basophiles, 10^9/L	5e-04 ±0.001	0.001 ±0.001
LUC, 10^9/L	0.01 ±0.002	0.01 ±0.002
Hemoglobin, mmol, L	5.3 ±0.1	5.1 ± 0.1
Hematocrit, L/L	0.3 ±0.01	0.3 ±0.05
MCV, ft	71.0 ±0.9	70.0 ±0.6
MCHC, mmol/L	19.8 ±0.8	19.9 ±0.1
Mean platelet volume, ft	11.6 ±0.5	11.0 ±0.2
Mean platelet count, g/L	227.9 ±2.3	226.0 ±2.4
Neutrophils, %	49.0 ±2.3	46.3 ±2.7
Lymphocytes, %	46.8 ±2.3	49.4 ±2.6
Monocytes, %	1.8 ±0.2	1.9 ±0.2
Eosinophils, %	1.7 ±0.2	1.8 ±0.3
Basophils%	0.08 ±0.01	0.15 ±0.07
LUC, %	0.5 ±0.1	0.4 ±0.1

*Values are mean ± SEM. MCV, mean corpuscular volume. There were no statistically significant differences in the variables ($p > 0.05$), except that rhIGF-1/BP-3 treatment increased leukocyte and neutrophil counts ($p < 0.05$). MCHC, mean corpuscular hemoglobin concentration LUC, large unstained cells.
