# Supplementary material for: Supplemental Insulin-Like Growth Factor-1 and Necrotizing Enterocolitis in Preterm Pigs
Source: Front Pediatr. 2021 Feb 4;8:602047. doi: 10.3389/fped.2020.602047 (PMC7891102; doi:10.3389/fped.2020.602047)
Supplement: Supplementary file 4 [file Table_4.pdf]

Supplementary table S4. Plasma biochemistry in 5 d-old preterm pigs treated with rhIGF-1/BP-3* or vehicle

**Parameter**	**rhIGF-1/BP-3**	**Controls**
Number of animals	20	21
Albumin g/l	11.7 ±0.8	11.1 ±0.5
Total protein g/l	28.3 ±0.8	27.9 ±0.6
Alkaline phosphatase, U/l	1862.6 ±173.8	1659.0 ±180.9
Alanine aminotransferase, U/l	28.8 ±8.8	22.5 ±7.5
Aspartate aminotransferase, U/l	37.5 ±11.1	28.8 ±6.6
Bilirubin, µmol/l	3.8 ±0.4	4.3 ±0.5
Cholesterol, mmol/l	1.9 ±0.1	1.8 ±0.1
Creatinine, µmol/l	56.2 ±1.8	58.1 ±1.6
Iron, µmol/l	9.5 ±1.3	9.3 ±0.8
Phosphate, mmol/l	1.8 ±0.1	1.8 ±0.1
Blood urea nitrogen, mmol/l	2.1 ±0.2	2.2 ±0.2
Gamma-glutamyltransferase, U/l	37.2 ±3.5	38.3 ±3.1
Calcium, mmol/l	3.3 ±0.03	3.3 ±0.03
Magnesium, mmol/l	0.9 ±0.02	0.8 ±0.02
Sodium, mmol/l	148.7 ±1.2	149.6 ±1.0
Potassium, mmol/l	5.3 ±0.4	5.1 ±0.3
Glucose, mmol/l	4.8 ±0.2	4.8 ±0.2
Creatinine kinase, U/l	128.2 ±29.8	104.6 ±26.8

*Values are mean±SEM. There were no statistically significant differences between the groups for any variable (all $p > 0.05$).
