# Supplementary material for: Supplemental Insulin-Like Growth Factor-1 and Necrotizing Enterocolitis in Preterm Pigs
Source: Front Pediatr. 2021 Feb 4;8:602047. doi: 10.3389/fped.2020.602047 (PMC7891102; doi:10.3389/fped.2020.602047)
Supplement: Supplementary file 5 [file Table_5.pdf]

Supplementary table S5. Macroscopic NEC scores of individual preterm 5 d-old pigs treated with rhIGF-1/BP-3 or vehicle*

Pig nr.	Treatment	Small intestinal NEC score (highest score of proximal, middle and distal regions)	Stomach NEC score	Colon NEC score (highest score of colon and caecum)
1	rhIGF-1/BP-3	1	3	3
2	rhIGF-1/BP-3	1	3	2
3	rhIGF-1/BP-3	1	1	1
4	rhIGF-1/BP-3	1	1	6
5	rhIGF-1/BP-3	1	1	1
6	rhIGF-1/BP-3	1	1	2
7	rhIGF-1/BP-3	1	1	4
8	rhIGF-1/BP-3	1	1	4
9	rhIGF-1/BP-3	1	3	3
10	rhIGF-1/BP-3	1	1	1
11	rhIGF-1/BP-3	1	1	6
12	rhIGF-1/BP-3	2	1	1
13	rhIGF-1/BP-3	1	3	3
14	rhIGF-1/BP-3	1	1	1
15	rhIGF-1/BP-3	1	1	3
16	rhIGF-1/BP-3	1	1	1
17	rhIGF-1/BP-3	3	3	3
18	rhIGF-1/BP-3	1	1	4
19	rhIGF-1/BP-3	3	1	1
20	rhIGF-1/BP-3	1	1	1
21	rhIGF-1/BP-3	1	1	4
22	rhIGF-1/BP-3	1	1	1
23	rhIGF-1/BP-3	3	1	3
24	rhIGF-1/BP-3	4	1	6
25	Control	1	3	4
26	Control	1	1	6
27	Control	5	5	5
28	Control	2	1	3
29	Control	1	1	3
30	Control	1	3	4
31	Control	1	5	6
32	Control	1	5	4
33	Control	2	6	4
34	Control	6	6	6
35	Control	1	1	1
36	Control	1	3	4
37	Control	6	1	5
38	Control	3	2	1
39	Control	1	1	4
40	Control	1	1	4

41	Control	1	1	1
42	Control	1	1	3
43	Control	2	1	4
44	Control	1	3	4
45	Control	1	1	4
46	Control	1	1	1
47	Control	2	1	1
48	Control	1	1	4

*NEC scores for individual pigs across gut regions: 1 = absence of lesions, 2 = local hyperaemia, 3 = hyperaemia, extensive edema and local haemorrhage, 4 = extensive hemorrhage, 5 = local necrosis and/or pneumatosis intestinalis, 6 = extensive necrosis and/or pneumatosis intestinalis. NEC was defined as a score of minimum 3, whereas severe NEC was defined as a score of minimum 4.
