# Supplementary material for: Supplemental Insulin-Like Growth Factor-1 and Necrotizing Enterocolitis in Preterm Pigs
Source: Front Pediatr. 2021 Feb 4;8:602047. doi: 10.3389/fped.2020.602047 (PMC7891102; doi:10.3389/fped.2020.602047)
Supplement: Supplementary file 6 [file Image_1.pdf]

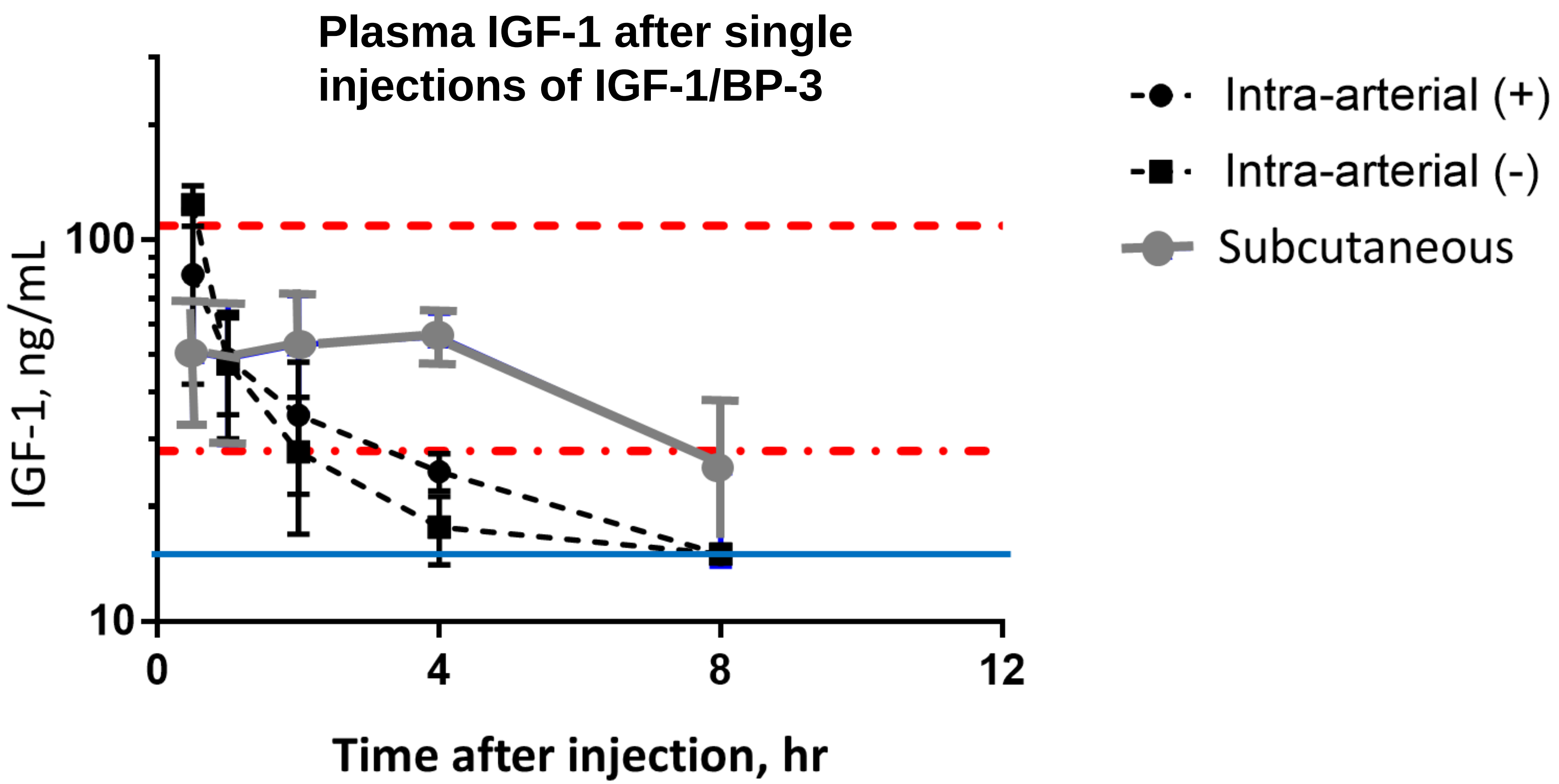

Supplementary Figure 1. Pharmacokinetics of IGF-1 in plasma of preterm pigs after intra-arterial administration, with (IA+) or without (IA-) maternal plasma immunization of piglets and subcutaneous administration (SC). The red dotted line indicates the desired physiological range. The blue line indicates baseline plasma IGF-1 levels (15 ng/mL) before injection.
